# Antimicrobial activities of endophytic fungi obtained from the arid zone invasive plant *Opuntia dillenii* and the isolation of equisetin, from endophytic *Fusarium* sp.

**DOI:** 10.1186/s12906-015-0722-4

**Published:** 2015-07-10

**Authors:** Pamoda B. Ratnaweera, E. Dilip de Silva, David E. Williams, Raymond J. Andersen

**Affiliations:** Department of Chemistry, University of Colombo, Colombo 03, Sri Lanka; Department of Chemistry and Earth, Ocean and Atmospheric Science, University of British Columbia, Vancouver, Canada; Department of Science and Technology, Uva Wellassa University, Badulla, Sri Lanka

**Keywords:** Endophytic fungi, Invasive plants, *Opuntia dillenii*, Equisetin, Arid ecosystem, *Fusarium* sp.

## Abstract

**Background:**

*Opuntia dillenii* is an invasive plant well established in the harsh South-Eastern arid zone of Sri Lanka. Evidence suggests it is likely that the endophytic fungal populations of *O. dillenii* assist the host in overcoming biotic and abiotic stress by producing biologically active metabolites. With this in mind there is potential to discover novel natural products with useful biological activities from this hitherto poorly investigated source. Consequently, an investigation of the antimicrobial activities of the endophytes of *O. dillenii,* that occupies a unique ecological niche, may well provide useful leads in the discovery of new pharmaceuticals.

**Methods:**

Endophytic fungi were isolated from the surface sterilized cladodes and flowers of *O. dillenii* using several nutrient media and the antimicrobial activities were evaluated against three Gram-positive and two Gram-negative bacteria and *Candida albicans*. The two most bioactive fungi were identified by colony morphology and DNA sequencing. The secondary metabolite of the endophyte *Fusarium* sp. exhibiting the best activity was isolated via bioassay guided chromatography. The chemical structure was elucidated from the ESIMS and NMR spectroscopic data obtained for the active metabolite. The minimum inhibitory concentrations (MICs) of the active compound were determined.

**Results:**

Eight endophytic fungi were isolated from *O. dillenii* and all except one showed antibacterial activities against at least one of the test bacteria. All extracts were inactive against *C. albicans*. The most bioactive fungus was identified as *Fusarium* sp. and the second most active as *Aspergillus* niger. The structure of the major antibacterial compound of the *Fusarium* sp. was shown to be the tetramic acid derivative, equisetin. The MIC’s for equisetin were 8 μg mL^−1^ against *Bacillus subtilis*, 16 μg mL^−1^ against *Staphylococcus aureus* and Methicillin Resistant *Staphylococcus aureus* (MRSA).

**Conclusions:**

*O. dillenii,* harbors several endophytic fungi capable of producing antimicrobial substances with selective antibacterial properties. By producing biologically active secondary metabolites, such as equisetin isolated from the endophytic *Fusarium* sp., the endophytic fungal population may be assisting the host to successfully withstand stressful environmental conditions. Further investigations on the secondary metabolites produced by these endophytes may provide additional drug leads.

## Background

Endophytic fungi are a diverse group of microorganisms that thrive asymptomatically in the healthy tissues of the host. Many of these endophytes are known to biosynthesis a plethora of bioactive secondary metabolites that may assist the host in protection and survival against pathogenic and insect attacks, stress tolerance and disease resistance [1, 2]. Moreover, some of these compounds have been proven to be useful as leads for novel drug discovery [3–5]. Thus, endophytes and their secondary metabolites not only play an important ecological role but also positively impact the field of medicine.

The arid zone ecosystem in South-East Sri Lanka is a small area extending only 20–35 Km inland to the north of Hambantota [6]. The area is characterized by limited rainfall of less than 1250 mm per year and mean annual temperatures of 27–30 °C with 5–7 months per year with a little or no rain [7]. A combination of dry sandy soils of high salinity and dry winds has made this environment unique and harsh when compared to other ecosystems in Sri Lanka. This area is mainly dominated by sedges, grasses, thorny shrubs including cactus species. Trees are less common in this arid zone ecosystem [6, 7]. The plants growing in this area are adapted to this harsh environmental setting.

Invasive plants are non-native plants introduced to a specific area and have a tendency to spread causing harm to the native biodiversity with consequent damage to the human economy and/or human health [8]. Invasive species are a growing concern in Sri Lanka [9]. *Opuntia dillenii* is a plant in the Cactaceae family introduced to Sri Lanka in the mid nineteenth century and has now become a well-established invasive plant in the Bundala National Park, a RAMSAR wetland site, in the South-Eastern arid zone of Sri Lanka, and is considered to be a national threat [10, 11]. *O. dillenii* has become highly competitive over the native plants in the area and has thus successfully adapted to both the abiotic and biotic stress conditions of the harsh environment. Since tolerance to biotic stress has been correlated with endophytic fungal natural products, it is likely that *O. dillenii* would be a rich source of endophytic fungi producing chemically diverse and biologically active secondary metabolites enhancing the host allelopathic effects and also providing protection against phytopathogenic microbes [12, 13]. However, investigations into the endophyte status of invasive plants in arid zone ecosystems, the secondary metabolites produced and the antimicrobial activities of these metabolites are limited. Therefore the antibacterial producing potential of endophytic fungi from *O. dillenii* collected in an unique ecological niche appeared to be an attractive target of investigation.

In this context, the objective of our study is to investigate the antimicrobial activities of the endophytic fungi from *O. dillenii* and to access for the potential production of bioactive secondary metabolites that may serve as leads for novel drug discovery. The current article reports the isolation of endophytic fungi from the invasive plant *O. dillenii* from a Sri Lankan arid zone, the antimicrobial properties of the organic extracts obtained, the bioassay-guided isolation and structure elucidation of the principal antimicrobial secondary metabolite along with the minimum inhibitory concentrations (MIC) observed against a number of pathogenic micro-organisms.

## Methods

### Isolation and antimicrobial screening of endophytic fungi

Healthy cladodes and flowers of the invasive plant *O. dillenii* were collected from the Bundala National Park in the South-East arid zone of Sri Lanka on July 2013. The samples were tightly sealed in polythene bags under humid conditions and kept at room temperature. The plant was identified using a detailed guide [14] and confirmed by comparing with the voucher specimen No. 12687 at the National Herbarium, Royal Botanical Garden, Peradeniya, Sri Lanka. The isolation of the fungal endophytes commenced within 24 hours of collection.

Prior to isolation of endophytes the plant material was surface sterilized. The cladodes and flowers were washed thoroughly with water for 10 and 3 minutes respectively, immersed in 70 % ethanol for 1–2 minute, 5.25 % Sodium hypochlorite for 2–5 minutes and again 70 % ethanol for 30–60 seconds [15]. The time used for the surface sterilization for each solvent used differed slightly depending on the texture of the plant material. To finish, the surface sterilized plant parts were washed with sterilized distilled water and allowed to dry inside a laminar flow cabinet. Small pieces of tissue were cut from the surface sterilized plant material and placed on dishes with potato dextrose agar (PDA), starch yeast peptone agar (SYP), yeast peptone dextrose agar (YPD), malt agar (ME) and malt peptone dextrose agar (MEA) media. The endophytic fungi that immerged from the tissues were transferred on to new PDA dishes and sequential sub culturing was done until pure cultures were obtained. Each pure fungal culture was grown on five freshly prepared PDA dishes and after 14–21 days depending on the growth of the fungus, the mycelium plus the medium was cut into small pieces and extracted with 200 mL of ethyl acetate for 24 hours. The ethyl acetate was filtered and the filtrate evaporated under reduced pressure.

The resulting residues were screened for antimicrobial activity against Gram positive *Bacillus subtilis* (UBC 344), *Staphylococcus aureus* (ATCC 43300), Methicillin Resistant *Staphylococcus aureus* (MRSA, ATCC 33591), Gram negative *Escherichia coli* (UBC 8161), *Pseudomonas aeruginosa* (ATCC 27853) and the pathogenic fungus *Candida albicans* (ATCC 90028) at 200 μg per disc using the agar disc diffusion method [16].

### Large scale culturing and extraction of endophytic fungi

Endophytic fungus that exhibited promising antimicrobial activity was cultured in 200 medium size Petri dishes (100 × 20 mm) of PDA for 17 days at room temperature. At the end of the incubation period the fungus together with the medium were cut into small pieces and immersed in 1 L of ethyl acetate for 48 hours and subsequently filtered through cotton wool. The extraction with ethyl acetate was repeated thrice. The filtrates were combined and the organic solvent evaporated under reduced pressure at room temperature. The resulting crude extract was weighed and screened for antimicrobial activity at 50 μg per disc to confirm the activity.

### Identification of the endophytic fungi

Colony morphological features of the two endophytic fungi with the most promising antimicrobial activity were recorded. Following this fungal DNA was extracted in the laboratory using a published protocol [17]. The extracted DNA was subjected to the polymerase chain reaction (PCR) using universal primers ITS1 and ITS4. Amplified DNA was sequenced and it was compared with existing DNA sequences in NCBI GenBank [18] to identify the fungi. PCR and DNA sequencing was done commercially.

### Fractionation, isolation and structure elucidation of the bioactive component

The principal bioactive component from the complex mixture of the crude extract of the major bioactive fungus, was obtained by bioassay guided chromatography. The crude extract (400 mg) was first fractionated on Sephadex LH-20 size exclusion column chromatography (3 × 115 cm) with methanol as eluent. The resulting fractions were combined according to the thin layer chromatography (TLC) profiles and the combined fractions were tested for antimicrobial activity using bioautography. The most active fraction (60 mg) was chromatographed on normal phase silica (3 × 20 cm column) with step-gradient elution (methanol : dichloromethane 1 : 99 to methanol). Finally the resulting bioactive fraction was purified by C_18_ reversed-phase high performance liquid chromatography (HPLC) using a semi-preparative column (0.94 × 25 cm) with 13:7 acetonitrile/(0.05 % trifluoroacetic acid (TFA)/water) as eluent to give 2 mg of pure compound.

The structure elucidation of the isolated compound resulted from analysis of the nuclear magnetic resonance (NMR) and mass spectral (MS) data obtained. ^1^H, ^13^C and 2D NMR data sets were obtained using a Bruker AVANCE 600-MHz spectrometer with a 5 mm cryoprobe with deuterated dimethyl sulfoxide, (DMSO-*d*_*6*_) as solvent. The electron spray ionization mass spectral (ESIMS) data was obtained using Bruker Esquire-LC electrospray mass spectrometer.

### Antimicrobial activity of the isolated pure compound

The bioactive compound was tested for antimicrobial activities against three Gram-positive bacteria, *B. subtilis* (UBC 344), *S. aureus* (ATCC 43300) and MRSA (ATCC 33591), two Gram-negative bacteria, *E. coli* (UBC 8161), *P. aeruginosa* (ATCC 27853) and the pathogenic fungus *C. albicans* (ATCC 90028). The minimum inhibitory concentrations (MICs) were determined using broth micro-dilution method according to National Committee for Clinical Laboratory Standards with modification using Mueller Hinton broth as the medium [19]. The commercial antimicrobial agents polymyxin B, rifamycin and amphotericin were used as positive controls.

## Results and discussion

### Isolation, antimicrobial activity and identification of endophytes

In total eight endophytic fungi, six from cladodes and two from flowers, were isolated from *O. dillenii*. The results of the antimicrobial tests of the ethyl acetate extracts of laboratory cultures of the eight fungi are listed in Table 1. Only one fungus, I8, was completely inactive. Three fungi, I4, I5, and I7 were active against only one organism tested while I3 and I6 were active against two test organisms. Most promising activities were shown by I1 and I2 which were active against three microorganisms each. None of the isolated endophytes were active against *C. albicans*.

**Table 1:** Antibacterial activities of the crude extracts of endophytic fungi isolated from *O. dillenii* at 200 μg/disc

Sample	Plant part used for the isolation		Diameter of the inhibition zone (mm)			
		*S. aureus*	MRSA	*B. subtilis*	*E. coli*	*P. aeruginosa*
I1	leaf	9	-	15	7	-
I2	flower	14	14	21	-	-
I3	leaf	-	-	11	-	9
I4	leaf	-	-	11	-	-
I5	leaf	-	-	-	-	9
I6	flower	8	-	12	-	-
I7	leaf	-	-	-	9	-
I8	leaf	-	-	-	-	-
+Ve		35	35	28	20	20
-Ve		-	-	-	-	-

+Ve control - Polymyxin B (30 μg/disc) for *P. aeruginosa*, *E. coli* and *B. subtilis*, Rifamycin (10 μg/disc) for *S. aureus*, MRSA and Amphotericin B (20 μg/disc) for *C. albicans*. -Ve control – Methanol

Some endophytic fungi have specific growth requirements and some species are not adapted to grow and sporulate in artificial culture media. Sometimes the fast growing fungal species outcompete the slower growing species [20]. Therefore use of several media with different nutrient aspects may probably be an advantage in isolating a large number of endophytic fungi including the cryptic species from the plant tissue. In this study four of the fungi were isolated in PDA medium while three and two were isolated in SYP and YPD media, respectively. Of the two most active fungi, I1 was isolated from both SYP and YPD media while I2 was isolated from SYP medium. This media specific isolation indicates that endophytic fungi look for specific nutrient needs or less competitive medium in order to spread their growth from the natural source to another.

The two endophytic fungi I1 and I2 were identified as *Aspergillus niger* and *Fusarium* sp. respectively. Endophytic *A. niger* showed the highest colonization in the cladodes of *O. dillenii* while the *Fusarium* sp. isolated from the pistil of the flowers showed the major biological activity. The isolation of bioactive compounds was carried out for the *Fusarium* sp. only.

**Fig. 1:**
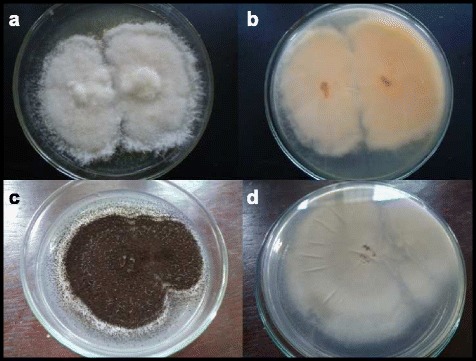
Endophytic **a** *Fusarium* sp. (*dorsal view*), **b** *Fusarium* sp. (*ventral view*), **c** *Aspergillus niger* (*dorsal view*) and **d** *A. niger* (*ventral view*) in culture

The *Fusarium* sp. had a white puffy mycelium with a peach colour pigment that secreted into the medium within seven to ten days of culture growth while *A. niger* produced black spores in a concentric ring pattern on PDA medium (Fig. 1). According to DNA sequence data and blast results obtained, these fungi showed 99 % identity to previously reported *Fusarium* sp. (GQ505759.1) and *A. niger* (JN561274.1) [21, 22]. In addition on the basis of 18S ribosomal RNA gene, partial sequence; internal transcribed spacer 1, 5.8S ribosomal RNA gene, and internal transcribed spacer 2, complete sequence and 28S ribosomal RNA gene, partial sequence, the major active fungi isolated in the current study were assigned to *Fusarium* sp. and *A. niger*.

There are a few reports of endophytic fungi and biological activities from other *Opuntia* species [23]. However other than for a preliminary survey of endophytic fungi from Australian samples of *O. dillenii* there are hardly any studies reporting the isolation procedures along with the identification of endophytic fungi and associated biological activities from *O. dillenii* [24]. Therefore to the best of our knowledge this is the first investigation to report the antibacterial activities of fungal endophytes of *O. dillenii* invasive plant.

### Isolation and structure elucidation of the active compound

**Fig. 2:**
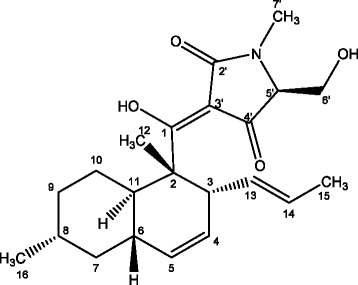
Equisetin-chemical structure

**Fig. 3:**
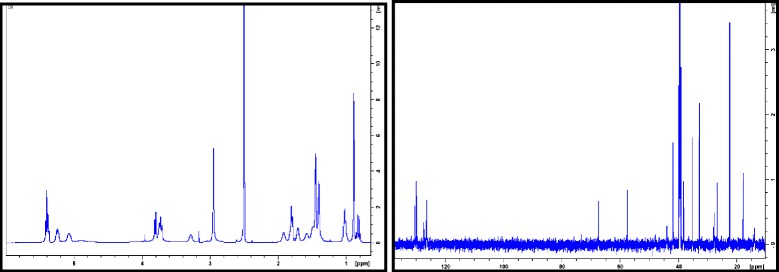
600 MHz ^1^H and ^13^C NMR spectra of equisetin in DMSO-*d*_*6*_

Large scale extraction of the *Fusarium* sp. with ethyl acetate gave 1 g of crude extract. Bioassay guided isolation of 400 mg of the crude extract resulted in the isolation of 2 mg of the active compound. The active component gave a [M + H]^+^ ion with m/z 374 in the low-resolution electrospray ionization mass spectrum. Analysis of ^1^H and ^13^C NMR data along with 2D NMR (COSY, HSQC, HMBC, ROESY) data revealed that the structure of the active compound matches that of the known tetramic acid derivative, equisetin (Fig. 2) [25] with a molecular formula of C_22_H_31_NO_4_ which was consistent with a molecular weight of 373 daltons as seen in the ESIMS. A comparison of ^13^C NMR values obtained in the present study for equisetin with those reported in the literature is shown in Table 2. The ^1^H and ^13^C NMR spectra of equisetin are illustrated in Fig. 3.

**Table 2:** Comparison of ^13^C NMR data of equisetin from the present study (in DMSO-*d*_*6*_) with published data (in CDCl_3_) [25]

C#	^13^C δ (ppm) for equisetin		C#	^13^C δ (ppm) for equisetin	
	Present study	Published values		Present study	Published values
1	190.2	190.6	12	14.0	13.7
2	47.9	48.4	13	127.3	127.1
3	44.1	44.6	14	130.4	130.4
4	126.4	126.2	15	17.8	18.2
5	129.8	129.8	16	22.4	22.5
6	38.2	38.4	2′	176.3	176.7
7	41.8	41.9	3′	100.6	99.8
8	33.0	33.3	4′	195.8	198.9
9	35.4	35.5	5′	67.5	66.4
10	27.8	28.1	6′	57.7	60.0
11	40.0	39.6	7′	26.8	27.2

Equisetin has previously been isolated from several species of *Fusarium* including *F. equiseti* from in which was the first report [26]. However this is the first report of equisetin from an endophytic *Fusarium* sp. isolated from *O. dillenii* flowers.

### Antimicrobial activity of equisetin isolated from endophytic *Fusarium* sp.

In the current study, as shown in Table 3, equisetin exhibited antibacterial activities against the Gram-positive bacteria *B. subtilis*, *S. aureus* and MRSA with MIC’s of 8–16 μg mL^−1^ and no activity against the Gram-negative bacteria *E.coli*, *P. aeruginosa* or pathogenic fungus *C. albicans.* In addition to the antibacterial activities equisetin has shown phytotoxicity and inhibited the *in vitro* recombinant integrase enzyme which is necessary for HIV replication [27].

**Table 3:** MIC values obtained for equisetin and the positive controls

	MIC values (μg mL^−1^)					
	*S. aureus*	MRSA	*B. subtilis*	*E. coli*	*P. aeruginosa*	*C. albicans*
Equisetin	16	16	8	-	-	-
Polymixin B	-	-	8	4	4	-
Rifamycin	0.015	0.015	-	-	-	-
Amphotericin	-	-	-	-	-	0.062

Previous research has reported that invasive plants’ competitive ability is enhanced by the production of secondary metabolites [28]. Yang *et al*. [29] and Alford *et al*. [30] have shown that secondary metabolites released from invasive plants directly inhibit seed germination of native plants while indirectly promoting the growth of invaders through the influence of nutrient cycling. Similarly Shipunov *et al*. has mentioned that in the host’s invaded range endophytes can increase the competitiveness of the host by producing metabolites inhibitory to evolutionarily native plants [31]. This view is also supported in the report by Aschehoug *et al*. [32].

In the context of these host/fungal relationships, compared to native plants, the production of the secondary metabolite equisetin by the *Fusarium* sp. isolated from the internal tissues of *O. dillenii*, may well enhance the competitive ability of this plant against microorganisms and perhaps increase its adaptability to withstand the harsh and biotic stress factors in its arid environment.

## Conclusions

This is the first study to describe the isolation and antibacterial activities of endophytic fungi from *O. dillenii*, an invasive plant from an arid zone ecosystem. The investigation has revealed that *O. dillenii* harbors several endophytic fungi which are capable of producing antimicrobial substances with selective antibacterial activities. The endophytic *Fusarium* sp. exhibited promising activity and the principal antimicrobial substance produced by this fungus proved to be the known secondary metabolite, equisetin. By producing such biologically active compounds, the endophytic fungal population may be assisting the host to successfully withstand stressful conditions and play a role in what can only be described as the successful spread of *O. dillenii* to the detriment of the native plants in the area. The findings of this study also suggests that endophytes from harsh and competitive environments have potential to be a productive source for the discovery of useful drug leads for innovative and improved pharmaceuticals.
